# The three-dimensionality of the hiPSC-CM spheroid contributes to the variability of the field potential

**DOI:** 10.3389/fphys.2023.1123190

**Published:** 2023-03-21

**Authors:** Minki Hwang, Su-Jin Lee, Chul-Hyun Lim, Eun Bo Shim, Hyang-Ae Lee

**Affiliations:** ^1^ AI Medic, Inc., Seoul, Republic of Korea; ^2^ Department of Predictive Toxicology, Korea Institute of Toxicology, Daejeon, Republic of Korea; ^3^ Department of Mechanical and Biomedical Engineering, Kangwon National University, Chuncheon, Republic of Korea

**Keywords:** hiPSC-CM, multi-electrode array, field potential, drug toxicity, simulation

## Abstract

**Background:** Field potential (FP) signals from human induced pluripotent stem cell-derived cardiomyocyte (hiPSC-CM) spheroid which are used for drug safety tests in the preclinical stage are different from action potential (AP) signals and require working knowledge of the multi-electrode array (MEA) system. In this study, we developed *in silico* three-dimensional (3-D) models of hiPSC-CM spheroids for the simulation of field potential measurement. We compared our model simulation results against *in vitro* experimental data under the effect of drugs E-4031 and nifedipine.

**Methods:** *In silico* 3-D models of hiPSC-CM spheroids were constructed in spherical and discoidal shapes. Tetrahedral meshes were generated inside the models, and the propagation of the action potential in the model was obtained by numerically solving the monodomain reaction-diffusion equation. An electrical model of electrode was constructed and FPs were calculated using the extracellular potentials from the AP propagations. The effects of drugs were simulated by matching the simulation results with *in vitro* experimental data.

**Results:** The simulated FPs from the 3-D models of hiPSC-CM spheroids exhibited highly variable shapes depending on the stimulation and measurement locations. The values of the IC_50_ of E-4031 and nifedipine calculated by matching the simulated FP durations with *in vitro* experimental data were in line with the experimentally measured ones reported in the literature.

**Conclusion:** The 3-D *in silico* models of hiPSC-CM spheroids generated highly variable FPs similar to those observed in *in vitro* experiments. The *in silico* model has the potential to complement the interpretation of the FP signals obtained from *in vitro* experiments.

## 1 Introduction

Drug toxicity screening is an important step in the development of new drugs. Especially, cardiotoxicity is related to arrhythmia which is often manifested by the prolongation of QT interval in the electrocardiogram (ECG) (Roden, 2016; Baracaldo-Santamaria et al., 2021). In the preclinical stage, the examination of cellular action potential (AP) under the effects of a drug using human induced pluripotent stem cell-derived cardiomyocytes (hiPSC-CMs) provides a clue for the proarrhythmic risk of the drug (Blinova et al., 2017; Yu et al., 2019). The *in vitro* models are often limited to two-dimensional (2-D) monolayers and lack myocardial cell–cell interactions, including heterotypic interactions between cardiomyocytes and cardiac fibroblasts (Kurokawa and George, 2016). With the recent advancements in bioengineering tools, three-dimensional (3-D) culture systems with varying degrees of complexity have gained significant traction in the field of drug testing and drug discovery (Fang and Eglen, 2017). Indeed, 3-D cultures of iPSC-CMs in the form of engineered myocardium have been shown to enhance physiological hypertrophy, improve maturity, and enhance drug response (Karbassi et al., 2020).

The comprehensive *in vitro* proarrhythmia assay (CiPA) (Vicente et al., 2018; Wallis et al., 2018), an international initiative for drug safety assessment includes the following three components in the preclinical stage: 1) *in vitro* assessment of drug effects on multiple cardiac ion channels, 2) *in silico* prediction of proarrhythmic risk using a computer model of human ventricular cardiomyocyte, and 3) *in vitro* assessment using hiPSC-CMs. For the *in vitro* assessment of drug effects on ion channels, patch clamp techniques are used to determine block potency such as half maximal inhibitory concentration (IC_50_) (Crumb et al., 2016; Mann et al., 2019). For the assessment using hiPSC-CMs, multi-electrode array (MEA) system is a platform that can characterize the electrical response of hiPSC-CMs under the effects of drugs (Harris, 2015; Andrysiak et al., 2021). While MEA measurements are high-throughput and less labor-intensive than patch clamp techniques, the output signal is field potential (FP) which is different from AP and requires working knowledge of the MEA measurement system to properly interpret the signal (Tertoolen et al., 2018; Kussauer et al., 2019). To detect electrical activities of tissues or organs *in vitro* and *in vivo*, the 3-D MEAs have emerged as a promising tool, but the 2-D MEAs are still widely used because there are many challenges to overcome (Choi et al., 2021; Demircan Yalcin and Luttge, 2021). Ideal 3-D MEA systems have to satisfy the requirements such as spatial coverage across the total volume of 3-D *in vitro* model, design flexibility according to types and sizes of models, a high spatial resolution to analyze the functional connectivity among cells in 3-D *in vitro* models, etc. (Shin et al., 2021). Moreover, the output signals from hiPSC-CMs on MEA system exhibit variability because there are different cell types in the population and the levels of differentiation are varied (Abbate et al., 2018). There have been a number of *in silico* studies that modeled and simulated MEA measurements on hiPSC-CMs with the purpose of complementing the interpretation of the *in vitro* signal (Tixier et al., 2017; Abbate et al., 2018; Raphel et al., 2018; Tertoolen et al., 2018). Most of those studies adopted 2-D model for the cells in the MEA well. Since many 3-D cardiac models are being developed as biomimetic models (Garzoni et al., 2009; Morrissette-McAlmon et al., 2020; Tani and Tohyama, 2022), the development and studies on the applicability of *in silico* 3-D models are needed to complement the interpretation of FP signals for these *in vitro* models. Because there are differences between 2-D and 3-D cultured hiPSC-CMs, the FP analysis from the 2-D model cannot be easily extrapolated to the 3-D model. Several studies report that 3-D cultured hiPSC-CMs have different physiological, contractile (Zhang et al., 2013; Gao et al., 2018; Silbernagel et al., 2020), and electrophysiological properties (Nunes et al., 2013; Daily et al., 2015; Lemoine et al., 2017; Correia et al., 2018) compared to 2-D cultured hiPSC-CMs.

The current study is an extension of those studies and we have developed an *in silico* 3-D model for the simulation of FP measurement of *in vitro* 3-D hiPSC-CM spheroids. Excitation wave propagation can occur toward or away from the recording electrode in the 3-D model which is simulated in this study by changing the initial locations of the excitation wave. We compared the simulation results with *in vitro* data under the effects of drugs E-4031 and nifedipine. The *in silico* 3-D model provides insights into the role of three-dimensionality in the variability of the FPs from hiPSC-CM spheroid, and has the potential to be used in the interpretation of *in vitro* data under the effects of drugs.

## 2 Methods

### 2.1 Formation of hiPSC-CMs spherical and discoidal models

Commercially available human iPSC-derived cardiomyocytes (Cardiosight®-S) were obtained from NEXEL Co., Ltd. (Seoul, Republic of Korea). Four differentiation batches for hiPSC-CMs (Lot# 12VAEZL, 12VVEZL, C2-6I1, C2-6U1) were used in this study. According to the COA (Certificate of Analysis) provided by the cell provider with the cell, all hiPSC-CM batches satisfied the provider’s QC criteria which are purity (>90% cTnT), MEA functionality, and drug reactivity for the reference drugs including E-4031 and nifedipine. The cells were cultured according to the instructions of the manufacturer. In brief, the cryopreserved hiPSC-CMs are thawed and seeded on the cell culture plate and then placed in an incubator at 37°C supplemented with 5% CO_2_. The cells were maintained for 1 week to induce time-dependent maturation and electrophysiological stabilization. After then, the monolayered hiPSC-CMs were dissociated and harvested with the STEMdiff™ Cardiomyocyte Dissociation Kit (Stemcell Technologies, Vancouver, Canada) and replated to a 3-D culture microwell (SpheroFilm, 500 μm inner diameter, INCYTO, Republic of Korea). The media in the microwell were changed daily, and the spheroids were cultured for 3 days. After 3 days, the hiPSC-CM spheroids were collected for MEA experiments. In the case of the discoidal models of hiPSC-CMs, the detached cells from culture plates on day 7 were directly plated onto the fibronectin pre-coated MEA plates to create a dome shape at a density of ∼70,000 plated cells per well.

### 2.2 MEA experiments

Before the sample plating on MEA plates, the recording areas of the 12-well MEA plate (Axion Biosystems, Atlanta, GA) were coated with human fibronectin (Sigma-Aldrich, St. Louis, MO) at 5 μL of 50 μg/mL in phosphate-buffered saline (PBS). Then, the plate was incubated at 37°C for 30 min. Then, the prepared hiPSC-CM spheroids were plated on the fibronectin pre-coated area of MEA plates. As previously mentioned, the dissociated and harvested cells from culture plates were directly plated on the MEA plates. Next, the MEA plates containing the hiPSC-CM spherical and discoidal models were incubated in a 37°C, CO_2_ incubator for 1 h to attach the sample to the multi-electrodes on each well. After 1 h, each well was filled with 1 mL of the culture medium. The medium was changed every 2 days thereafter for 3–7 days to obtain the spherical and discoidal models of hiPSC-CMs with spontaneous and synchronous electrical activity on the MEA plates.

**FIGURE 1:**
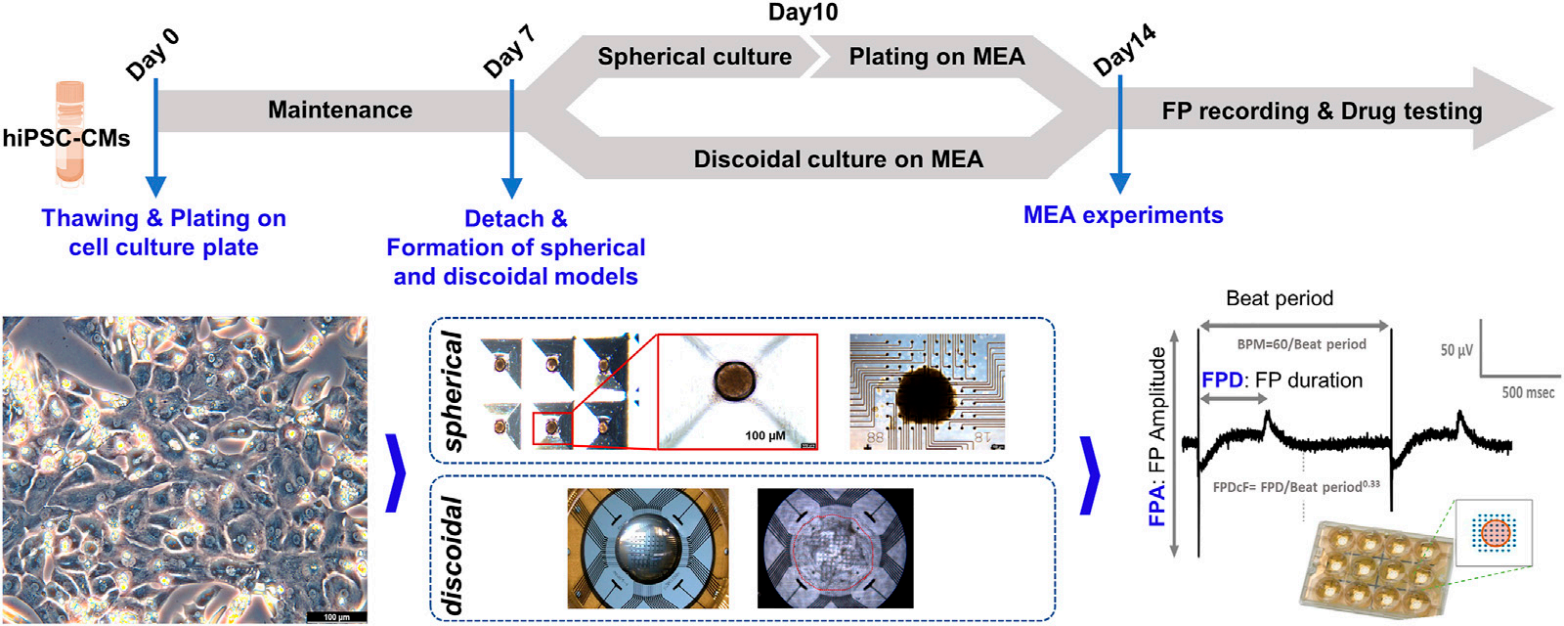
Experimental procedures of MEA assay. Experimental procedures are shown for the formation of spherical and discoidal hiPSC-CM models and MEA assay.

To evaluate the drug responses of the hiPSC-CM spheroids for ion channel-specific blockers, the effects of E-4031 (for a blocker of the hERG channel currents) and nifedipine (for a blocker of the calcium channel currents) on cardiac FPs of the hiPSC-CM spheroids were studied. The full of media on the plates was changed at least 4 h before recordings. FPs were recorded from spontaneously beating hiPSC-CM spheroids. For the FP recording, the MEA plate was placed on the Maestro MEA system (Axion Biosystems, GA, USA) keeping 37°C and 5% CO_2_ conditions. Data were filtered with a Butterworth 0.1–2 kHz band-pass filter. The drugs were prepared with 1000X in dimethyl sulfoxide (DMSO) of the final target concentration and treated in each well as a cumulative method from the lowest concentration to the highest concentration every 20 min. The FP waveforms of the last 1 min of each dose were recorded and analyzed for data analysis. The beat per minute (BPM), FP amplitude (FPA), and FPD_cF_ (FP duration (FPD) corrected with Fridericia’s formula: FPD_cF_ = FPD/Beat Period^0.33^) were analyzed with the AxIS program (Axion Biosystems, ver. 2.3.2). Experimental procedures for forming the models and field potential recording are shown in Figure 1.

### 2.3 Scanning electron microscope (SEM) image acquisition

SEM imaging and analysis for hiPSC-CMs spherical and discoidal models was conducted at Korea Research Institute of Bioscience and Biotechnology (KRIBB) according to the internal protocol. In brief, the samples were fixed in a 2.5% paraformaldehyde-glutaraldehyde mixture buffered with 0.1 M phosphate (pH 7.2) for 2 h, postfixed in 1% osmium tetroxide in the same buffer for 1 h, dehydrated in graded ethanol, and substituted by isoamyl acetate. Then they were dried at the critical point in CO_2_. Finally, the samples were sputtered with gold in a sputter coater (SC502, POLARON) and observed using the scanning electron microscope, FEI Quanta 250 FEG (FEI, USA) installed in KRIBB.

**FIGURE 2:**
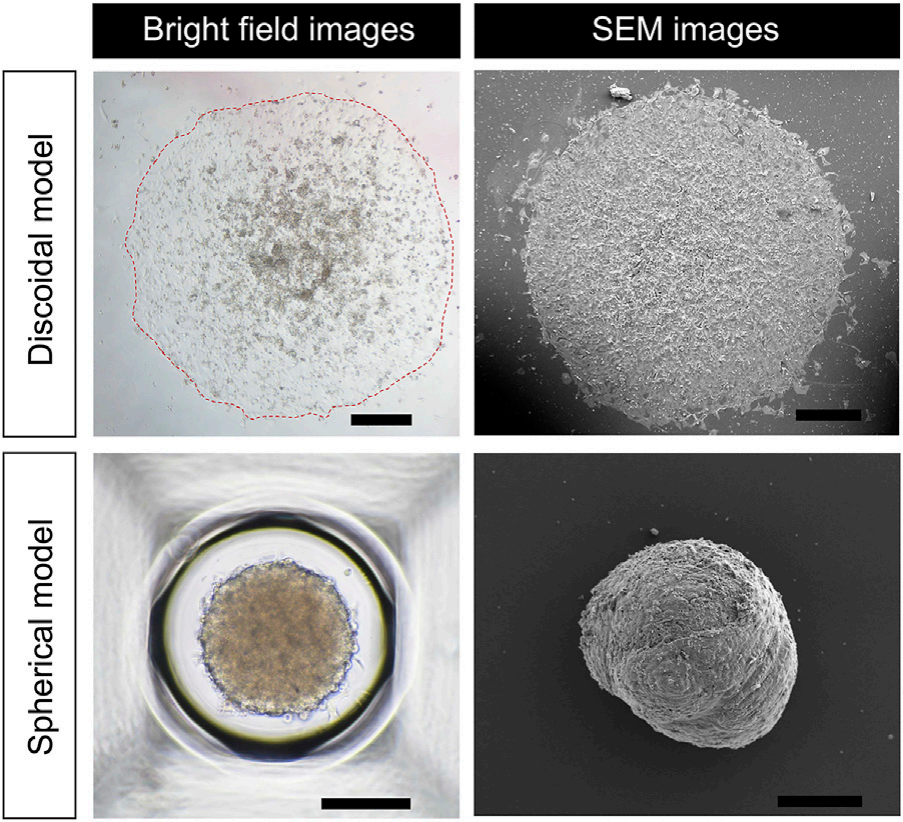
Morphology of discoidal and spherical hiPSC-CM models. Bright field and SEM images of hiPSC-CM models in discoidal and spherical shapes are shown. Scale bar in discoidal images, 500 μm; scale bar in spherical images, 200 μm.

Figure 2 shows optical micrographs of the discoidal and spherical models of hiPSC-CMs. The discoidal model has a diameter of approximately 1876.29 ± 64.59 µm (*n* = 3 from two batches) and is attached flatly to the bottom surface with a slightly thicker dome-like shape in the middle. In the case of the spherical models, it has a three-dimensional round shape and a diameter of 318.34 ± 10.25 µm (*n* = 15 from two batches). In both models, adjacent cells are overlapped and tightly attached to conduct electrical signals (Figure 2).

### 2.4 *In silico* 3-D models of hiPSC-CM spheroids

*In silico* 3-D models of hiPSC-CM spheroids were constructed in spherical and discoidal shapes (Figure 2). For the spherical model, it was slightly deformed such that a flat surface was generated, which was in contact with the bottom surface of the well of the MEA device. The diameters of the models were set to 1 and 2 mm for spherical and discoidal models, respectively, which were consistent with experimental observation. Tetrahedral meshes were generated inside the models for the computation of AP propagation. The number of meshes was increased until the simulation results converged. The propagation of the AP in the model was obtained by numerically solving the following monodomain equation:

$$\frac{\partial V_{m}}{\partial t}=\nabla ∙D\nabla V_{m}-\left(I_{ion}+I_{stim}\right)\frac{1}{C_{m}}$$

where *V*_m_ is the membrane potential, *t* is time, *D* is the diffusion coefficient, *I*_ion_ and *I*_stim_ are ionic and stimulation currents, respectively, and *C*_m_ is the membrane capacitance. The diffusion coefficient was set to 0.0072 mm^2^/s which resulted in the experimentally observed conduction velocity of 0.12 ± 0.0028 mm/ms. The equation was numerically solved by using finite element method (Im et al., 2008). For the calculation of *I*_ion_, the O’Hara-Rudy dynamic (ORd) human ventricular cell model was used (O'Hara et al., 2011). At the locations of the electrodes, the extracellular potential was calculated as described in a study (Ugarte et al., 2014). The method uses the ratio of the extracellular to intracellular conductivities which we set to three following the value used by Abbate et al. (2018). The FP measurement system was modeled as an equivalent circuit with an electrode capacitance, an electrode resistance, and the internal resistance of the measurement device as described in previous studies (Moulin et al., 2008; Raphel et al., 2018). The FP was calculated as the electric current through the equivalent circuit due to the extracellular potential, multiplied by the internal resistance of the measurement device (Raphel et al., 2018).

### 2.5 Simulation of the effect of drug

The effects of drugs E-4031 and nifedipine were incorporated by partly blocking *I*_Kr_ and *I*_CaL_ channels, respectively, more with increasing drug concentration. The percentages of each channel blockade were determined such that the simulated FPD were matched with experimentally measured data. Using the percentages of each channel blockade at multiple drug concentrations, the IC_50_ and Hill Coefficient were calculated by applying the Hill equation (Goutelle et al., 2008).

### 2.6 Statistical analysis

All experimental results were statistically processed using GraphPad Prism (GraphPad, San Diego, United States), AxIS program (Axion Biosystems, ver. 2.3.2), and Excel (Microsoft, Redmond, WA, United States). All values are represented as mean ± Standard error of mean (SEM) and n represents the number of experimental replicates with individual samples. Statistical significance was determined using the Student’s *t*-test; *p* < 0.05 was considered to indicate statistical significance.

## 3 Results

### 3.1 Analysis of field potentials from discoidal and spherical models of hiPSC-CMs

**FIGURE 3:**
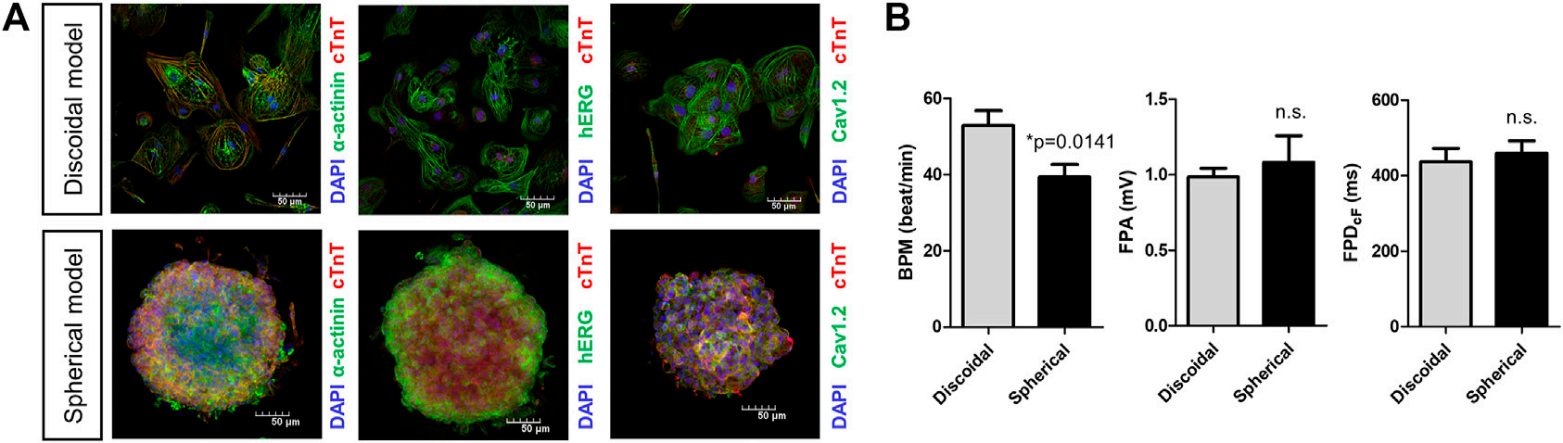
Characterization of spherical and discoidal hiPSC-CM models. **(A)** Immunocytochemistry images for cardiac myogenic markers (cTnT, a-actinin) and ion channels (Nav1.5, Cav1.2). **(B)** Comparison of field potential parameters for spherical and discoidal hiPSC-CM models. *p < 0.05 by one-way ANOVA with Student’s *t*-test (*n* = 15 for the discoidal models, *n* = 9 for the spherical models, each from two differentiation batches). n.s., not significant; BPM, beats per minute; FPA, field potential amplitude; FPD_cF_, FP duration (FPD) corrected with Fridericia’s formula to correct the FPD dependence on beating rate.

Field potentials were recorded in the hiPSC-CMs-based discoidal and spherical models using the MEA system. To check the quality of the hiPSC-CMs used in model production, we confirmed that cardiac troponin T (*cTnT*) and α-cardiac actin (*α-actinin*) were expressed in >90% of hiPSC-CMs. Also, it was confirmed that the human ether-à-go-go-related gene (*hERG*) and cardiac L-type calcium channel (*Cav1.2*), which contribute to ventricular repolarization and are the leading causes of drug-induced cardiac arrhythmia in drug development, were also expressing in the cell membrane (Figure 3A). The field potential parameters were recorded and analyzed by attaching discoidal or spherically shaped multi-cellular hiPSC-CMs on the MEA plates. The average BPM was significantly higher in the discoidal model than the spherical model [52.8 (*n* = 15) vs. 39.3 bpm (*n* = 9), *p* = 0.0141]. The field potential amplitudes were 0.99 and 1.1 mV for the discoidal and spherical models, respectively, and there was no statistical difference between the two groups. In the case of the FPD_cF_ as well, there was no statistical difference between the two groups with the values of 436.4 and 459.2 ms for the discoidal and spherical models, respectively (Figure 3B).

### 3.2 Simulated field potentials from 3-D spheroid models

**FIGURE 4:**
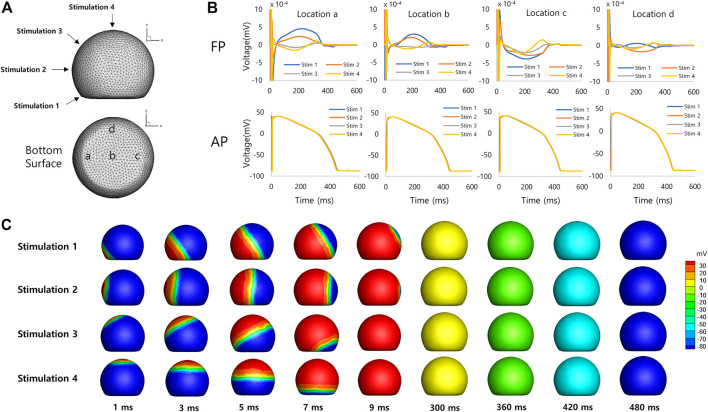
Spherical model of hiPSC-CM spheroid. **(A)** Geometry of the spherical model of hiPSC-CM spheroid with stimulation (1, 2, 3, and 4) and measurement (a, b, c, and d) locations. **(B)** Simulated field potentials and action potentials for different stimulation and measurement locations. The difference between FPD (314 ± 92 ms, mean ± SD, *n* = 16, SD = Standard Deviation) and APD (427 ± 4 ms, mean ± SD, *n* = 16) is 113 ms based on mean values. **(C)** Simulated action potential propagations for different stimulation sites.

Figure 4 shows simulated FPs obtained from the spherical model of hiPSC-CM spheroid. The electrical stimulations were applied at four different sites on the model surface from the bottom to the top of the model (Figure 4A). The simulated FPs obtained at four different electrode locations at the bottom surface (a, b, c, and d in Figure 4A) are shown in Figure 4B. The shape of the signal exhibits a large variation similar to the observation reported by Abbate et al. (Abbate et al., 2018). The signals showed inverted shape between the electrode locations a and c for the stimulation sites 1 (blue color). The amplitudes of the signals were relatively small at the electrode location d compared to the other locations. The positive deflection corresponding to the repolarization was relatively more noticeable for the stimulation sites near the top of the model. Figure 4C shows the AP propagation on the surface of the model for each stimulation site.

**FIGURE 5:**
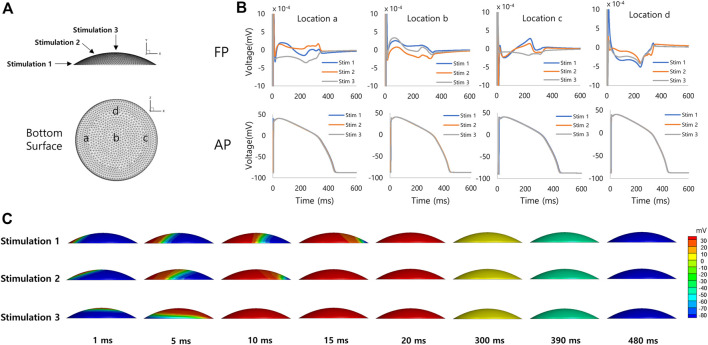
Discoidal model of hiPSC-CM spheroid. **(A)** Geometry of the discoidal model of hiPSC-CM spheroid with stimulation (1, 2, and 3) and measurement (a, b, c, and d) locations. **(B)** Simulated field potentials and action potentials for different stimulation and measurement locations. The difference between FPD (292 ± 46 ms, mean ± SD, *n* = 12) and APD (426 ± 4 ms, mean ± SD, *n* = 12) is 134 ms based on mean values. **(C)** Simulated action potential propagations for different stimulation sites.

Figure 5 shows simulated FPs obtained from the discoidal model of hiPSC-CM spheroid. The shape of the signal showed a large variation as in the case of the spherical model. The positive deflection corresponding to the repolarization was relatively more noticeable at the electrode location c for the stimulation sites 1 and 2 (Figures 5A, B). Figure 5C shows the AP propagation on the surface of the model for each stimulation site.

### 3.3 The effect of drug simulated using the 3-D spheroid model

**FIGURE 6:**
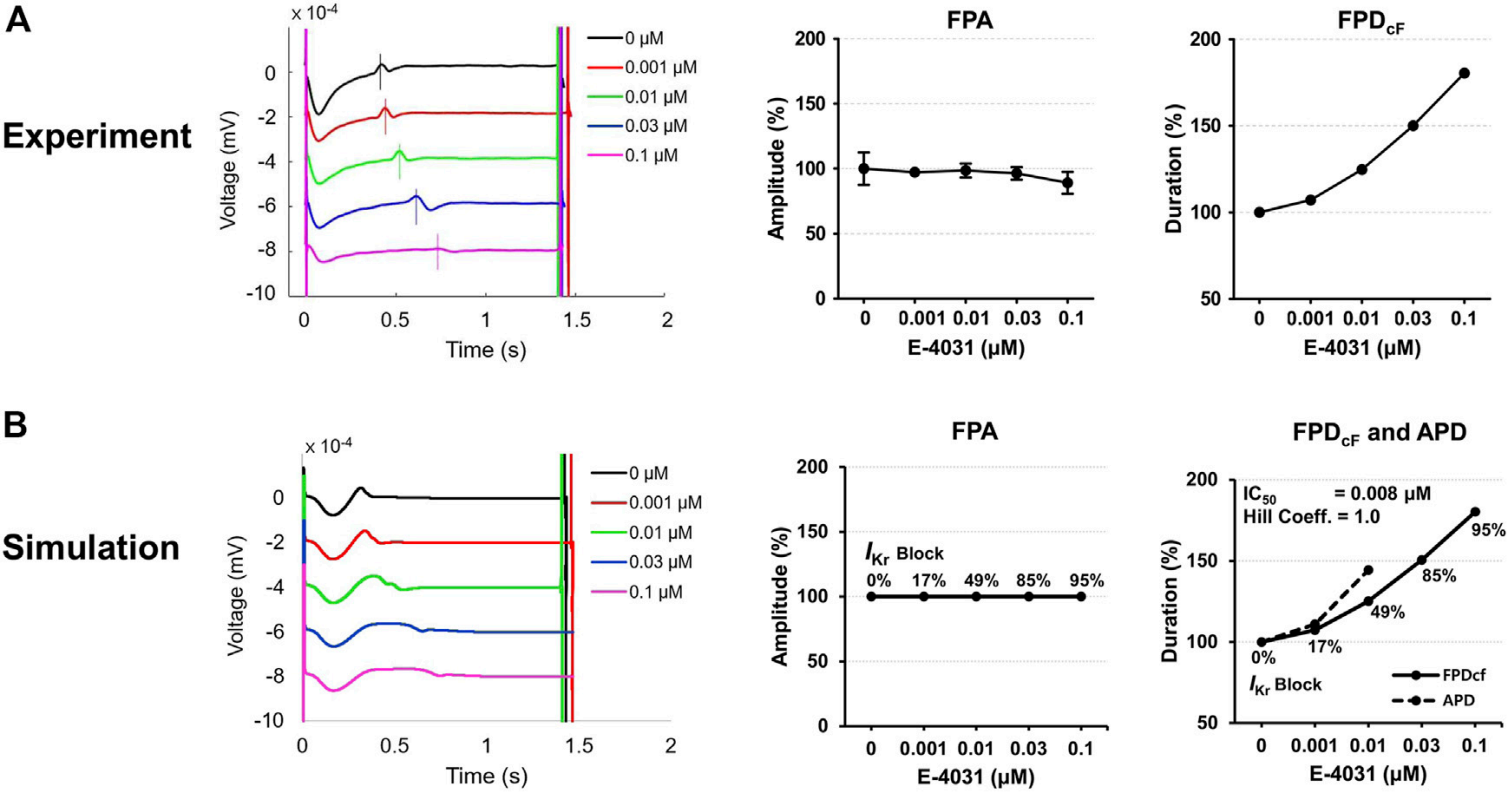
Comparison of field potentials between *in vitro* experiment and *in silico* simulation for E-4031. **(A)** Effect of E-4031 on the FPs in the experimental model (*n* = 12 from two differentiation batches). **(B)** Effect of E-4031 on the FPs in the simulation model. The APDs at 0.03 and 0.1 μM are not available due to the failure of AP repolarization (Supplementary Figure S1). The shapes, amplitude, and heart rate corrected duration of field potentials are compared. BPM, beats per minute; FPA, field potential amplitude; FPD_cF_, FP duration corrected with Fridericia’s formula. FPD_cF_ = FPD/(beat period)^0.33^.

Figure 6 shows experimentally measured and simulated FPs under the effect of E-4031 from the spherical model of hiPSC-CM spheroid. The use of the mid-myocardial cell model, for which the action potential duration (APD) is longer than endocardial or epicardial cell, resulted in similar FPD as the experimentally observed one before the drug was applied. The simulated APs are shown in Supplementary Figure S1. To match the experimentally observed FPD increase with increasing E-4031 concentration, the percentage of the *I*_Kr_ blockade was searched for each E-4031 concentration used in the *in vitro* experiment because E-4031 is an hERG channel blocker. The *I*_Kr_ had to be blocked 95% when E-4031 concentration was 0.1 μM, for which the FPD_cF_ increased by 80% (Figure 6). The IC_50_ calculated using the percentages of the *I*_Kr_ blockade at each E-4031 concentration was 8 nM which is close to the experimentally determined value of 7.7 nM with a Hill coefficient of 1.0 reported in the literature (Zhou et al., 1998). The simulated positive deflection corresponding to the repolarization tended to be flattened with increasing drug concentration which was also observed in the study of Tertoolen et al. (Tertoolen et al., 2018) although the flattening was not observed in our *in vitro* experiment. The simulated FPA change with increasing drug concentration was negligible, which qualitatively agreed with experimental data (Figure 6).

**FIGURE 7:**
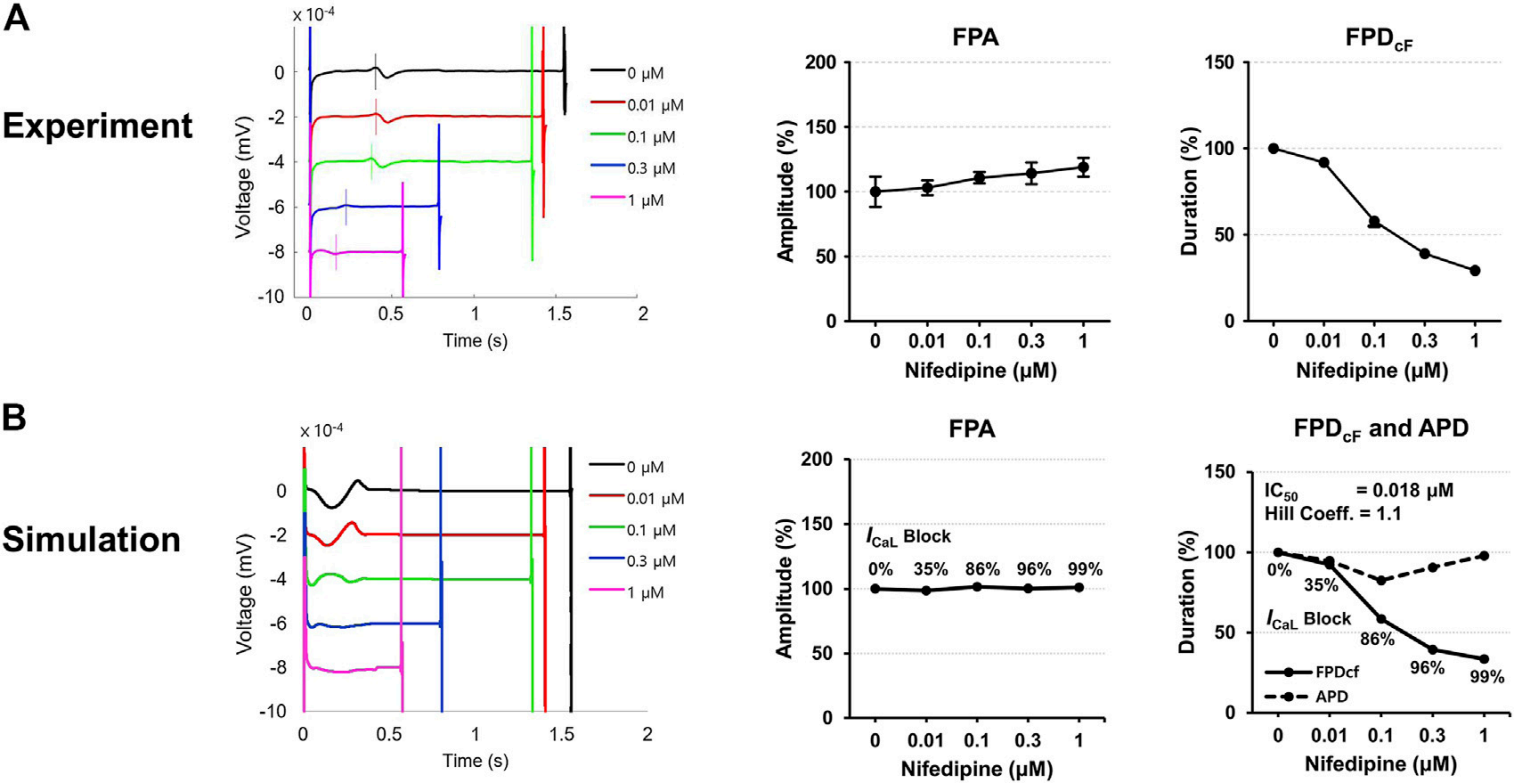
Comparison of field potentials between *in vitro* experiment and *in silico* simulation for nifedipine. **(A)** Effect of nifedipine on the FPs in the experimental model (*n* = 11 from two differentiation batches). **(B)** Effect of nifedipine on the FPs in the simulation model. The shapes, amplitude, and heart rate corrected duration of field potentials are compared. BPM, beats per minute; FPA, field potential amplitude; FPD_cF_, FP duration corrected with Fridericia’s formula. FPD_cF_ = FPD/(beat period)^0.33^.

Figure 7 shows experimentally measured and simulated FPs under the effect of nifedipine for the spherical model of hiPSC-CM spheroid. The simulated APs are shown in Supplementary Figure S2. Because nifedipine is a Ca^2+^ channel blocker, the percentage of the *I*_CaL_ blockade was searched for each nifedipine concentration used in the experiment to match the experimentally observed FPD change with increasing nifedipine concentration. The IC_50_ calculated using the percentages of the *I*_CaL_ blockade at each drug concentration were 0.018 μM which is close to the experimentally measured value of 0.012 μM with a Hill coefficient of 1.02 reported in the literature (Kramer et al., 2013). The simulated FPA change with increasing drug concentration was negligible, although experimental data showed slightly increasing FPA with increasing drug concentration (Figure 7).

## 4 Discussion

For the safety assessment of new drugs in the preclinical stage, utilization of hiPSC-CMs on MEA system is a relatively new platform and was adopted in the CiPA initiative. While MEA system has technical advantages over patch clamp measurement, the output signals from hiPSC-CMs on MEA system are highly variable and specific knowledge of the MEA system is needed for proper interpretation of the signal. In this study, we developed 3-D *in silico* models of hiPSC-CM spheroid on MEA system and validated the model using experimentally measured FP signals under the effect of drugs. The discoidal model has a 2.5-D dome-like shape with a diameter of approximately 1.8 mm and the spherical models has a 3-D round shape and a diameter of approximately 300 µm (Figure 2). Recent studies have shown that large 3-D spheroids (>500 µm) have a large necrotic core compared to small spheroids (<350 µm) and low drug effect and dye uptake (Gaze et al., 1992; Buffa et al., 2001; Horas et al., 2005; Gong et al., 2015; Daster et al., 2017; Singh et al., 2020; Han et al., 2021).

There have been *in silico* studies in which the variability of the FP signals was modeled using various approaches. Abbate et al. (2018) modeled the variability of the FP by introducing different cell phenotypes in the model and rescaling the amplitudes of the AP after observing that their two-dimensional model generated relatively homogeneous signals. They also tested different distributions of the cell types in the model and reported that the clustered configuration produced a higher level of variability in the FP shapes. Tixier et al. (2017) applied a probabilistic approach in distributing different cell types in the model. They assumed that the transition states between the two cell types were determined by a random process parameter and the cells in transition states were distributed using a correlation function. In our 3-D study, the variability of the FPs was observed depending on the stimulation sites and electrode locations even when a single cell type was present in the model. This implies that the three-dimensionality of the spheroid on MEA system contributes to the variability of the FPs although other factors such as different cell types and the level of differentiation are definite sources of the heterogeneity. Examining the effects of the heterogeneity in action potentials among the cells is the next step of this study. The variation of the AP traces was less noticeable than that of FP as observed in the 2-D study by Abbate et al. (2018). The small difference in the voltage distribution in the spheroid caused by different initial activation site may contribute to the large variation in FP shapes. More study seems to be needed to clarify the role of three-dimensionality in the variability of the FPs from hiPSC-CM spheroid.

For the simulation of the effect of drug using the present model of MEA system, the change of the FPD based on the repolarization wave was examined in this study, which was adopted in many *in vitro* and *in silico* studies (Tixier et al., 2017; Raphel et al., 2018; Kussauer et al., 2019). The repolarization wave of FP signal corresponds to the end of repolarization of the AP (Tertoolen et al., 2018), and the change of the APD correlates with that of QT interval in the ECG (Hwang et al., 2019). A drawback of the approach used in this study is that the percentage of ion channel block depending on the concentration of a drug cannot be known *a priori*, and it only can be determined by matching the change of FPD with experimental data. As a result, *in vitro* assessment of drug effects on ion channels should be performed first, which is the number one step of the CiPA initiative (Vicente et al., 2018). As *in silico* prediction of APD change under the effects of a drug is performed using *in vitro* data of ion channel block in the second component of the CiPA initiative, the *in silico* approach presented in this study also needs the *in vitro* data of ion channel block. However, the present *in silico* approach could be used in the interpretation of the experimental data of MEA measurement by comparing the data of ion channel block such as IC_50_ and Hill coefficient. Also, the present approach could provide insight into the characteristics of the spheroid such as the composition and distribution of different cell types.

There are some limitations in the present study. Due to the large variability of the FP signal, it was not easy to establish a correlation between FPD and APD. The FP signal shape was dependent on the locations of stimulation and measurement. The FP signal also turned out to be sensitive to the change in the slope of AP in the 2D study by Abbate et al. (2018). Accordingly, it seems that caution should be taken in identifying the repolarization peak in the FP signal. In the cases of higher concentrations of the two drugs tested in this study, the FP repolarization peaks did not mean AP repolarization (Supplementary Figures S1, S2). In the cases of higher concentrations of E-4031, the FP repolarization may have resulted from more or less the same transmembrane potential (around 0 mV) over the entire spheroid due to the failure of AP repolarization. In the cases of higher concentrations of nifedipine, the FP repolarization peaks were hardly recognizable, and the peaks appeared to be far from the AP repolarization. These suggest that FP signals should be interpreted with great caution, especially at higher concentrations of drugs, and further investigation is needed in this regard. The cellular electrophysiological model used is not hiPSC-CM model, and single cell type was implemented in the spheroid models. Incorporation of recently developed electrophysiological models of hiPSC-CM (Paci et al., 2013; Koivumaki et al., 2018) would provide simulation results closer to the *in vitro* data. Also, the spherical and discoidal shapes are simplified models of the 3-D spheroids. Nevertheless, the present 3-D models of hiPSC-CM spheroid provide insights into the role of three-dimensionality in the variability of the FPs from hiPSC-CM spheroid, and have potential to be used in the interpretation of *in vitro* data under the effects of drugs.

## Data Availability

The original contributions presented in the study are included in the article/Supplementary Material, further inquiries can be directed to the corresponding authors.
