# Supplementary material for: The three-dimensionality of the hiPSC-CM spheroid contributes to the variability of the field potential
Source: Front Physiol. 2023 Mar 21;14:1123190. doi: 10.3389/fphys.2023.1123190 (PMC10070703; doi:10.3389/fphys.2023.1123190)
Supplement: Supplementary file 1 [file DataSheet1.PDF]

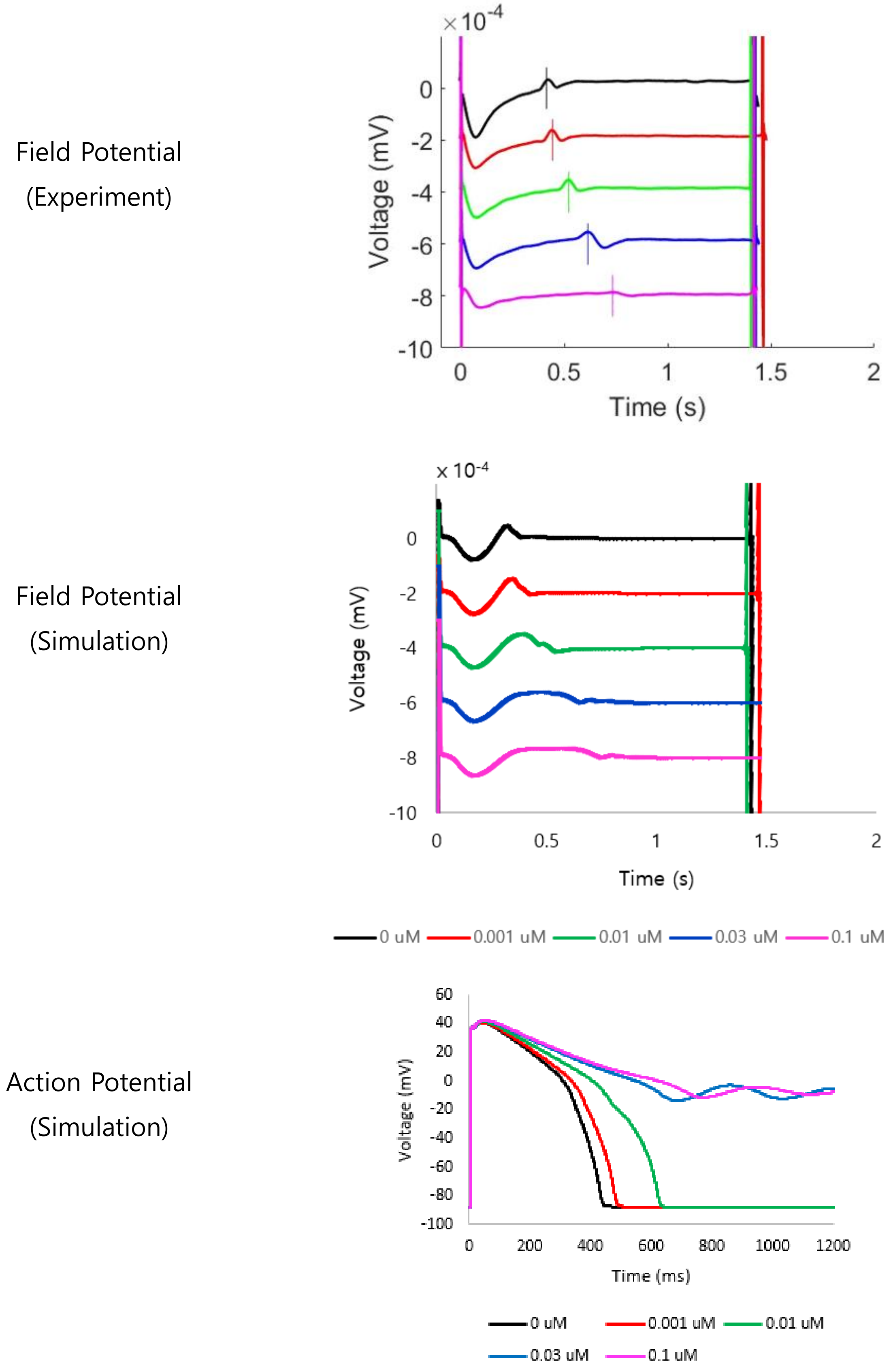

Figure S1. Comparison of field potential and action potential under the effect of E-4031.

Field Potential
(Experiment)

Field Potential
(Simulation)

Action Potential
(Simulation)

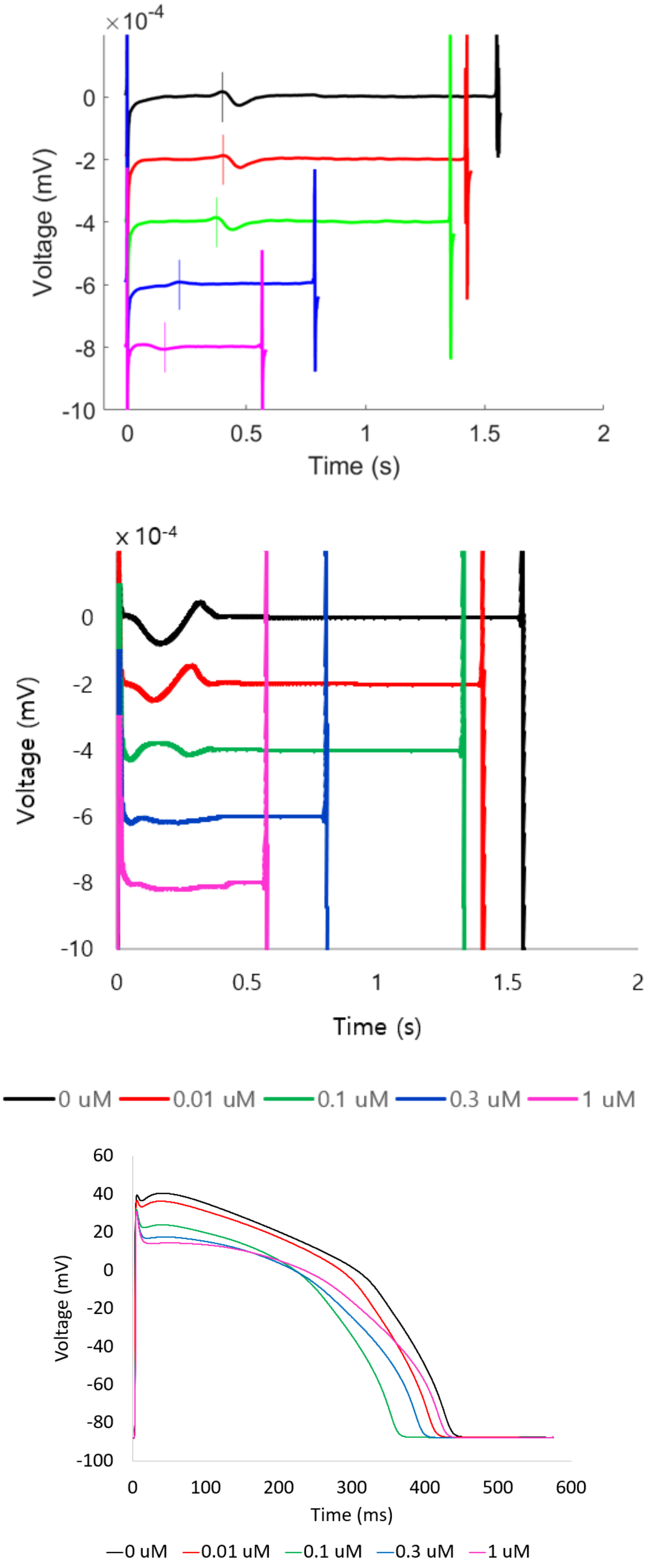

Figure S2. Comparison of field potential and action potential under the effect of nifedipine.
